# How do we decide what to do? Resting-state connectivity patterns and components of self-generated thought linked to the development of more concrete personal goals

**DOI:** 10.1007/s00221-016-4729-y

**Published:** 2016-07-21

**Authors:** Barbara Medea, Theodoros Karapanagiotidis, Mahiko Konishi, Cristina Ottaviani, Daniel Margulies, Andrea Bernasconi, Neda Bernasconi, Boris C. Bernhardt, Elizabeth Jefferies, Jonathan Smallwood

**Affiliations:** 1 Department of Psychology, York Neuroimaging Centre, University of York, Heslington, York, UK; 2 Neuroanatomy and Connectivity Group, Max Planck Institute for Human and Cognitive Brain Sciences, Leipzig, Germany; 3 Neuroimaging of Epilepsy Lab, Montreal Neurological Institute, McGill University, Quebec, Canada; 4 Neuroimaging Laboratory, IRCCS Santa Lucia Foundation, Rome, Italy

**Keywords:** Mind-wandering, Goals, Future thought, Hippocampus

## Abstract

Human cognition is not limited to the available environmental input but can consider realities that are different to the here and now. We describe the cognitive states and neural processes linked to the refinement of descriptions of personal goals. When personal goals became concrete, participants reported greater thoughts about the self and the future during mind-wandering. This pattern was not observed for descriptions of TV programmes. Connectivity analysis of participants who underwent a resting-state functional magnetic resonance imaging scan revealed neural traits associated with this pattern. Strong hippocampal connectivity with ventromedial pre-frontal cortex was common to better-specified descriptions of goals and TV programmes, while connectivity between hippocampus and the pre-supplementary motor area was associated with individuals whose goals were initially abstract but became more concrete over the course of the experiment. We conclude that self-generated cognition that arises during the mind-wandering state can allow goals to be refined, and this depends on neural systems anchored in the hippocampus.

## Introduction

Although acting in the moment satisfies many of our primary needs, important achievements—such as constructing the pyramids or landing on the moon—involve the capacity to represent things that *could* be the case, allowing the generation of a series of steps that can make imagined scenarios come about. Several important positive and negative outcomes have been linked to elements of experience (Kane et al. 2007), behaviour (Flehmig et al. 2007) and neural functioning (Smith et al. 2015) that are independent of environmental input; nevertheless, the real-world significance of the different aspects of the cognition that we generate in our idle moments remains largely unexplored.

One adaptive value we might derive from the capacity to self-generate thought unrelated to the task in hand is the chance to make progress on problems that we cannot act on immediately. As a species, we devote a substantial amount of our free time to thinking about the future: experience-sampling studies have identified a prospective bias in naturally occurring thoughts across many different cultures (Baird et al. 2011; Stawarczyk et al. 2011; Song and Wang 2012; Ruby et al. 2013). Although these studies show that future-oriented thought is common during the mind-wandering state, they do not explain whether this leads to more effective plans and if so what mechanism underlies this change. It is possible that prospective thoughts could help refine strategies for achieving personal goals, a process that would be adaptive if it allowed future actions to be specified in greater detail (Gollwitzer and Sheeran 2006). Prior work has shown inconsistent evidence that tasks that encourage stimulus-independent thought increase the chance that a novel solution to an old problem will be generated through a process of incubation (Baird et al. 2012; Smeekens and Kane 2016); however, as incubation is assumed to be largely unconscious (Smith and Blankenship 1991), this does not explain the benefits we may gain from devoting conscious thought to a problem. The current experiment examined the hypothesis that future-focussed thought during mind-wandering helps us generate the steps we should take to achieve important personal goals.

 We also hoped to understand the structure of spontaneous neurocognitive activity that underpins the process through which people make plans for the future. The medial temporal lobe system, and in particular the hippocampus, is a plausible candidate neural system for allowing us to form more concrete plans for the future. Lesion studies and functional imaging work have documented that the hippocampus is important in forming new memories with high levels of episodic detail (Eichenbaum 1993; Aggleton and Brown 1999), as well as the retrieval of these memories in the service of constructing imagined scenes (for reviews, see Buckner and Carroll 2007; Schacter et al. 2007; Hassabis and Maguire 2009). In both contexts, the hippocampus forms a cohesive network with other regions such as the ventromedial pre-frontal cortex that bind together different elements of scenes to form holistic representations of episodes (Marr et al. 1991; McClelland et al. 1995; Dickerson and Eichenbaum 2010). The role of the hippocampus in making predictions using imagination is known as the *prospective consolidation hypothesis,* emphasising that prior experiences are crystallised in long-term memory with the aim of making better predictions of what may happen (Buckner 2010; Smallwood 2013). By providing a mechanism that encodes events into memory, as well as generating simulations of what may occur in the future based on representations of what has already happened, the hippocampus could be important in developing descriptions of personal goals in the absence of being able to immediately act upon them.

**Fig. 1:**
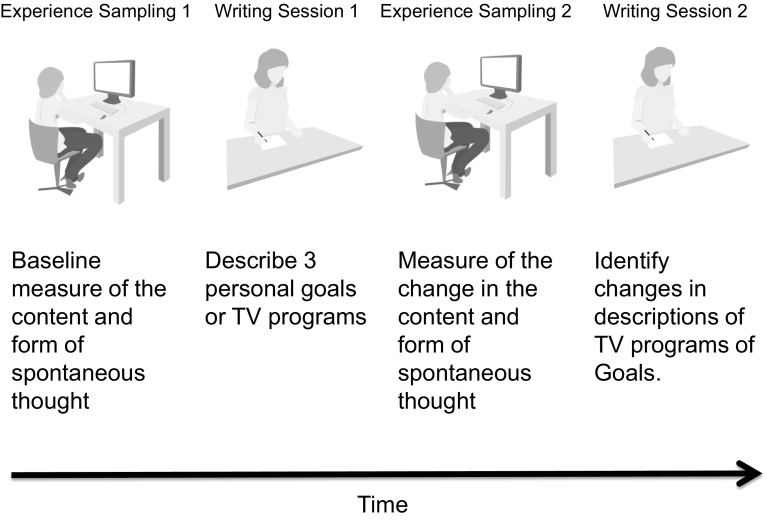
Experimental protocol for the behavioural session. Participants performed a single laboratory session with four stages. The first stage was an externally focused task in which the content and form of their thoughts that occur during the mind-wandering state was assessed using experience sampling, providing a baseline measure of experience for each participant. Next, they were allocated to one of two conditions: (1) to write about either three personal goals or (2) to write about three of their favourite TV programmes for 15 min. Following this they repeated the cognitive task including the experience-sampling measures allowing the assessment of how experience changed. Finally, participants repeated the writing task

The current study examined the experiential and neural basis of the capacity to transform personal goals into a series of concrete steps that can generate specific actions and compared this with the development of descriptions generated from memory but *not* linked to goals (descriptions of familiar TV programmes). We chose TV programmes as control since it would provide memorial information that was familiar but not personally relevant. We used resting-state functional magnetic resonance imaging (fMRI) to characterise the intrinsic architecture of the hippocampus in a sample of healthy participants. In a subsequent laboratory session, participants first completed a baseline session of an undemanding task in which the contents of the thoughts that emerge during mind-wandering was assessed. Next, they were allocated to spend 15 min either writing descriptions of three personal goals, or three TV programmes on a computer. After completing this section of the experiment, the participants performed a second session of the mind-wandering task. Finally, participants repeated the written description task for goals or TV programmes that they had performed previously. We measured the content and form of the thoughts that emerged during mind-wandering using multidimensional experience sampling (MDES, see Smallwood et al. 2016). The written transcripts were rated along a number of dimensions including their temporal focus, their emotional qualities and their levels of concrete detail. This design is described schematically in Fig. 1.

We examined two aspects of the data relevant to how we specify our goals using imagination. First, if the tendency to engage in prospective thought during mind-wandering is implicated in the generation of more concrete goals, then during the mind-wandering task, future thinking should change in tandem with beneficial changes in goal descriptions; for example, there might be increases in the level of detail that these goals contain (Gollwitzer and Sheeran 2006). Second, if this tendency to refine personal goals depends on a brain network that includes the hippocampus, then individual differences in goal descriptions should predict its functional connectivity profile; for example, the strength of its connection to regions involved in action selection or decision-making. In contrast, trait variability in the strength of functional connectivity between hippocampus and areas implicated in the control of action should not predict individual differences in the ability to specify greater detail about TV programmes.

## Methods

### Participants

One hundred participants (26 males, age mean = 20 years; age SD = 1.72) were recruited using an Electronic Experiment Booking System at the University of York. Approval was granted by the Ethics Committee of the Psychology Department and York Neuroimaging Centre. In a subset of participants (*n* = 42, 13 males, age mean 20.4 years, age SD = 1.72, 23 participated in the goals condition), high-resolution functional and structural MRI data were available (obtained on average 3 days before the behavioural testing study). The investigation was in accordance with the Declaration of Helsinki, and all participants provided written informed consent. Participants received a payment of £20 or a commensurate amount of course credits for participation. Participants who participated in the neuroimaging element of the experiment received the same level of compensation for participating in this additional experimental session. We decided on the sample size for this experiment based on prior studies exploring similar questions (e.g. Baird et al. 2013; Ruby et al. 2013). Participants were recruited separately for the imaging and behavioural session. In this study, we also measured heart rate and blood pressure; however, these data will be reported elsewhere. We report how we determined our sample size, all data exclusions (if any), all manipulations and all measures in the study.

### Behavioural session

#### Experimental procedure

The procedure of the behavioural session is presented schematically in Fig. 1. It began with a session in which participants completed a cognitive task to provide a baseline measure of the content and form of spontaneous thought. Next, participants were allocated to write for 15 min on either three of their most important personal goals or to write for a similar period on three of their favourite TV programmes. After this period, all participants repeated the cognitive task allowing the content and form of spontaneous thought to be assessed on a second occasion. Finally, they were asked to write about the same topics allowing us to determine any changes in their representations of TV programmes or personal goals that had occurred over the delay period.

#### Cognitive task

We assessed experience in a variant of the task used by Konishi et al. (2015). The task alternates between a 0-back and a 1-back condition and was employed to provide a measure of experience during both the baseline and the experimental phases of the experiment. In both conditions, participants viewed different pairs of shapes (non-targets) appearing on the screen divided by a vertical line; the pairs could be: a circle and a square, a circle and a triangle, or a square and a triangle for a total of 6 possible pairs. In both tasks, a block of non-targets was followed by a target requiring participants to make a manual response. The target consisted of a small stimulus presented at the middle of the vertical line in either blue or red and the colour was counterbalanced across participants. In the 0-back condition, the target was flanked by one of two shapes and participants had to indicate by pressing the appropriate button which shape matched the target shape. In the 1-back condition, the target was flanked by two question marks and participants had to respond depending on which side the target shape was on the previous trial. Responses were made using the left and right arrow keys. Each block lasted between 40 and 120 s before switching to the other condition; the change of condition was signalled by a message (“SWITCH”) that remained on screen for 5 s. On each trial, the number of non-targets preceding the targets varied between 2 and 6, the number of trials per block varied between 2 and 5 and the total number of blocks was 8 for each condition. The order of conditions was counterbalanced across participants, and each session of the task lasted approximately 20 min. The total number of targets was 15–20 per condition per session (0-back and 1-back). Presentation rate of stimuli was jittered: fixation crosses ranged from 2 to 4 s in steps of 0.1 s; non-targets varied from 1 to 3 s in steps of 0.1 s. Targets were presented for a maximum of 4 s and were terminated by the participant’s response.

#### Multidimensional experience sampling (MDES)

The content of thought was measured using MDES: a technique that measures multiple different elements of experience as it occurs during the mind-wandering state with a specific focus on the patterns of covariance within the observed data (Ruby et al. 2013; Ruby et al. 2013; Engert et al. 2014; Gorgolewski et al. 2014). Participants were interrupted randomly during the task by thought probes asking participants a series of questions on the content of their experience in the moment immediately prior to the interruption. Participants received an average of 6 probes in each condition in each session. As is standard in our work (e.g. Ruby et al. 2013; Engert et al. 2014), we randomised the number of probes to ensure that a particular probing regime could influence the extent of mind-wandering that was reported. The questions were administered in a quasi-random order with the first question always about task focus, followed by mini-blocks of questions examining the extent to which their thoughts involved future events, past events, themselves, or other people, images or words, and whether their thoughts were intrusive, vague and non-specific or detailed, and positively or negatively valanced. On each occasion, the order of each mini-block as well as the order of questions within each mini-block was randomised. Participants could rate their thoughts on a scale from 0 (completely off-task) to 9 (completely on-task), except for the emotional content where they had to rate their thoughts on a scale between two extremes, from negative to positive. This procedure is consistent with prior studies using this approach (Ruby et al. 2013; Engert et al. 2014).

#### Writing task

Participants were randomly assigned to either the goals or TV program condition. In the goal condition, 51 participants were asked to choose three important personal goals in life. In the TV program condition, 49 choose three of their favourite TV programmes. In both the pre- and post-sessions, each group was given 15 min to write about the allocated topic. Participants wrote their answers in a word document and were encouraged to use all of the time to write about their assigned topic. They were asked not to worry about punctuation or grammar. Participants were encouraged to be as sincere as possible. To facilitate an honest description of goals, participants were told that they did not need to provide identifying information. For example, in the goal condition participants were told that if they were to write about a goal that involved a friends or relative they did not need to provide their real name. The participants were also reassured that their data would be treated anonymously.

At the end of the experiment, participants were asked what they thought the aim of the experiment was. Sixty-five percentage of them thought it was something related to how TV programmes/goals affected thought content, performance or concentration, 18 % thought it was about mind-wandering/day-dreaming, 1 % thought it was on personality, 1 % on depression and mental health and 15 % had no idea. Importantly, no participants indicated that they expected to repeat the task of writing about either the TV programmes or their goals. Finally, participants were compensated for their participation.

### Transcript analysis

Two independent raters that were blind to experimental condition, the thoughts the participants reported during the study, the results of the functional connectivity analysis, and the aim of the experiment analysed transcripts from both groups. Raters were provided with the descriptions in Table 1 and were asked to rate the transcripts for each type of content on a Likert scale from 1 (not at all) to 5 (completely). To assess the reliability of the different rates, we calculated Cohen k which provides a good estimate of the degree to which two raters make consistent estimates of the same phenomenon (Cohen 1960). It represents the proportion of agreement corrected by chance, and these are presented in Table 2. The average score for each category from the two raters was calculated and used in the subsequent analyses.

**Table 1:** Categories for the analysis of the transcripts

Category	Description
Future	Refers to the future (e.g. uses the future tense)
Past	Refers to the past (e.g. uses the past tense)
Self	Refers to him/herself (e.g. uses first person pronoun)
Other	Refers to other people (e.g. family, friends)
Positive	The content is positive (e.g. expresses positive emotions)
Negative	The content is negative (e.g. expresses negative emotions)
Concrete	Focus on specific actions (e.g. a study schedule) or objects (e.g. money, books)
Abstract	Focus on general goals (e.g. success) or abstract terms (i.e. freedom, equity)

**Table 2:** Mean (SD) for the ratings of the transcripts for the two sessions

	Session 1		Session 2		Agreement (Cohen)
	Mean	SD	Mean	SD	
Past	2.0	0.6	2.0	0.6	.70
Future	2.1	1.1	2.2	1.1	.96
Positive	3.0	0.8	2.9	0.8	.90
Negative	2.2	0.6	2.3	0.6	.77
Self	3.0	1.0	3.0	1.0	.95
Other	2.8	0.8	2.9	0.9	.78
Abstract	3.1	0.7	3.1	0.6	.77
Concrete	3.0	0.7	3.0	0.5	.68

### Neuroimaging session

In a subset of participants (*n* = 42, 13 males, age mean 20.4 years, age SD = 1.72, 23 participated in the Goals condition), high-resolution functional and structural MRI data were available (obtained on average 3 days before the behavioural testing study). All MRI data were acquired at the York Neuroimaging Centre (YNIC) using a GE 3.0 Tesla Signa Excite HDx scanner using an eight-channel phased-array head coil. The session began with a 9-min resting-state fMRI scan, in which participants were asked to focus on a fixation cross. The blood oxygen-level-dependent (BOLD) fMRI time-series with fat saturation were acquired using a 9-min gradient single-shot echo planar imaging (EPI) sequence [repetition time (TR) = 3000 ms, echo time (TE) = 19 ms, flip angle = 90°, matrix = 64 × 64, field of view (FOV) = 192 mm, 60 slices with interleaved (bottom-up) acquisition order, slice thickness = 3 mm, voxel size = 3 × 3 × 3 mm, 180 volumes]. Subsequently, we acquired high-resolution structural scans for each of the participants using an isotropic 3D fast spoiled gradient-recalled echo (3D FSPGR) T1-weighted sequence (TR = 7.8 ms, TE = 3 ms, flip angle = 20°, matrix = 256 × 256, 176 sagittal slices with 1 mm thickness, voxel size = 1.13 × 1.13 × 1 mm). To facilitate co-registrations, a high-resolution T1-weighted in-plane anatomical picture was also acquired for all participants, using a fluid-attenuated inversion recovery (T1 FLAIR).

### fMRI pre-processing

All fMRI pre-processing and analyses were performed using FSL. We extracted the brain from the skull using the BET toolbox for both the FLAIR and the structural T1-weighted images, and these scans were registered to standard space using FLIRT (Jenkinson and Smith 2001). Prior to conducting the functional connectivity analysis, the following pre-statistics processing was applied to the resting-state data; motion correction using MCFLIRT (Jenkinson et al. 2002); slice-timing correction using Fourier-space time-series phase shifting; non-brain removal using BET (Smith 2002); spatial smoothing using a Gaussian kernel of FWHM 6 mm; grand-mean intensity normalisation of the entire 4D dataset by a single multiplicative factor; high-pass temporal filtering (Gaussian-weighted least-squares straight line fitting, with *σ* = 100 s); Gaussian low pass temporal filtering, with *σ* = 2.8 s.

### First-level analysis

Seed regions were based on anatomical masks of the left and right hippocampus in MNI152 space, derived from a recently published open-access data repository (Kulaga-Yoskovitz et al. 2015). Average time-series of these masks were extracted and used as explanatory variables in a subject-wise functional connectivity analysis, which also included the following nuisance regressors: the first five principal time-series components extracted from white matter (WM) and cerebrospinal fluid (CSF) masks in accordance with the CompCor method (Behzadi et al. 2007) and six motion parameters. WM and CSF masks were generated by segmenting each individual’s high-resolution structural image (using FAST in FSL). The default tissue probability maps, referred to as prior probability maps (PPMs), were registered to each individual’s high-resolution structural image (T1 space), and the overlap between these PPM and the corresponding CSF and WM maps was identified. Finally, these maps were thresholded (40 % for the SCF and 66 % for the WM), binarized and combined. The six motion parameters were calculated in the motion-correction step during pre-processing. Linear displacements in each of the three Cartesian directions (*x*, *y*, *z*) and rotations around three axes (pitch, yaw, roll) were included for each individual. No individual exceeded xyz-displacements >1 mm and rotations beyond 1°. No global signal regression was performed (Murphy et al. 2009).

## Results

### Behavioural performance

A mixed analysis of variance (ANOVA) compared performance (accuracy and RT) on the cognitive task used during MDES. This analysis had two within-participant factors each with two levels: task (0-back/1-back) and session (first/second). Experimental group (goals/TV programmes) was included as a between participants variable. With respect to accuracy, the analysis revealed a main effect of task [*F* (1, 97) = 20.1, *p* < .001] with lower accuracy in the 1-back (mean = .92, SE = .01) than in the 0-back (mean = .97, SE = .01). Analysis of RT revealed a main effect of task [*F* (1, 97) = 110.9, *p* < .001] with slower responses in the 1-back (mean 1.08 s, SE = .02) than in the 0-back (mean = .98 s, SE = .02) and an effect of session [*F* (1,97) = 5.6, *p* < .05] with longer response times in the first session (mean = 1.01 s, SE = .02) than in the second (mean = 0.98 s, SE = .02). It also revealed a task by session by group interaction [*F* (1, 97) = 8.5, *p* < .01]. We calculated the change in RT from the first to the second session in each task and compared these scores across experimental groups using a MANOVA. This yielded an effect of experimental group on RT in the 0-back task [*F* (1, 97) = 5.42, *p* < .05] but no effect on RT in the 1-back task (*p* < .1). In the TV programmes condition, response times in the 0-back task were shorter in the second session [mean = .91 s (SE = .02)] than in the first [mean = .97 (SE = .03), *t* (47) = 2.36, *p* < .05].

We used principal components analysis (PCA) with varimax rotation in SPSS to decompose the dimensionality of both the experience-sampling data and the written descriptions.

#### Written descriptions

**Fig. 2:**
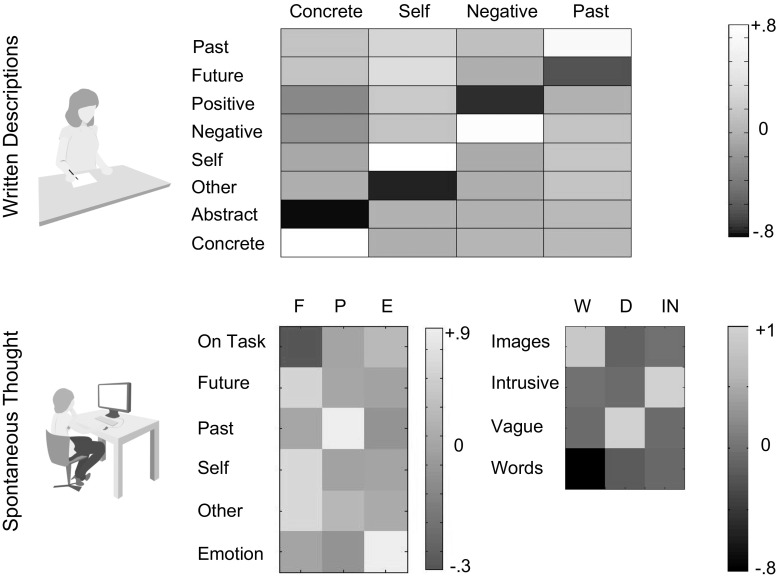
Results of the decomposition of the written descriptions and experience-sampling data. Heat maps illustrating the results of decompositions using principal components analysis (PCA) for both the written descriptions (*upper panel*) and the experience-sampling data (*lower panel*). In both cases, varimax rotation was applied to the subsequent solutions. *F* Future, *P* past, *E* emotion, *W* words/images, *D* detail, *IN* intrusive

Table 2 presents the average ratings for the transcripts in each session. We calculated the change between initial and subsequent ratings for each participant for each dimension, creating a single vector that describes how different aspects of the participants’ descriptions changed over the course of the experiment. Applying PCA to this vector yielded four components that are presented as a heat map in the upper panel of Fig. 2. Factor solutions were selected with an eigenvalue >1. Component 1 describes changes in the level of concrete details of the description, accounting for approximately 23 % of the variance and with a positive weighting reflecting increases in concreteness.^1^ Component 2 reflects an increased emphasis on the self versus other people in the descriptions and accounted for 21 % of the overall variance. Component 3 reflects an increased emphasis on negative rather than positive aspects of the descriptions for 18 % of the variance. Component 4 describes a change in the emphasis of the past relative to the future in the descriptions and accounts for 13 % of the overall variance. Subsequently, we projected these data back into subject space, allowing us to describe each individual in terms of how much they changed their descriptions along the dimensions that the decomposition procedure identified.

#### Multidimensional experience sampling

Table 3 presents the data from the experience-sampling probes separated by session. To ensure comparability with the prior investigation, we decomposed these data at the trial level, concatenating the data from each probing episode for each individual into a single matrix and applying PCA with varimax rotation. A decomposition with three orthogonal factors yielded solutions that are broadly consistent with prior investigations (Ruby et al. 2013; Engert et al. 2014) (see lower left-hand panel of Fig. 2): (a) *Future focused thoughts* with a high weighting on thoughts about the self in the future, accounting for 36 % of the observed variance, (b) *Past focused thoughts* with a high weighting on thoughts about self and others in the past, accounting for 18 % of the variance and (c) *Task-related thoughts* with a high weighting indicating positive task focused experiences and a low weighting indicating negative off-task experiences, accounting for 18 % of the variance.

**Table 3:** Mean (SD) for the multidimensional experience sampling (MDES)

Question	Session 1		Session 2	
	Mean	SD	Mean	SD
Task	.61	.24	.53	.27
Future	.43	.28	.46	.28
Past	.31	.25	.37	.28
Self	.51	.27	.54	.26
Other	.39	.29	.46	.29
Emotion	.57	.18	.59	.18
Images	.49	.27	.52	.27
Words	.58	.28	.57	.26
Intrusive	.36	.25	.37	.26
Detail	.43	.27	.44	.27

Our decomposition of the form of thoughts yielded three components, which can be seen in the left panel of Fig. 2: (a) *The modality of the thoughts* (images or words) with a high weighting reflecting thoughts that were described as words, accounting for 37 % of the variance, (b) *The level of intrusiveness in the thoughts* with a high weighting indicating more intrusive thoughts, accounting for 25 % of the variance and (c) *The level of detail* in the thoughts, with a low weighting indicating a higher level of detail, accounting for 25 % of the overall variance. The loadings for each component describing the content and form of thought are presented as heat maps in the lower right-hand panel of Fig. 2, and these components are consistent to a pattern revealed through similar decomposition process in two different data sets (Smallwood et al. 2016). For the purpose of analysis, the six components describing the content and form of spontaneous thoughts were averaged across the two MDES sessions (i.e. recorded before and after the initial description task, respectively) for each participant. Finally, a change score was calculated by subtracting the component in the first MDES session from the component in the second MDES session. These data correspond to changes from baseline in particular aspects of spontaneous thought following the description task.

#### Relation between the change in descriptions and spontaneous thought during the mind-wandering task

**Fig. 3:**
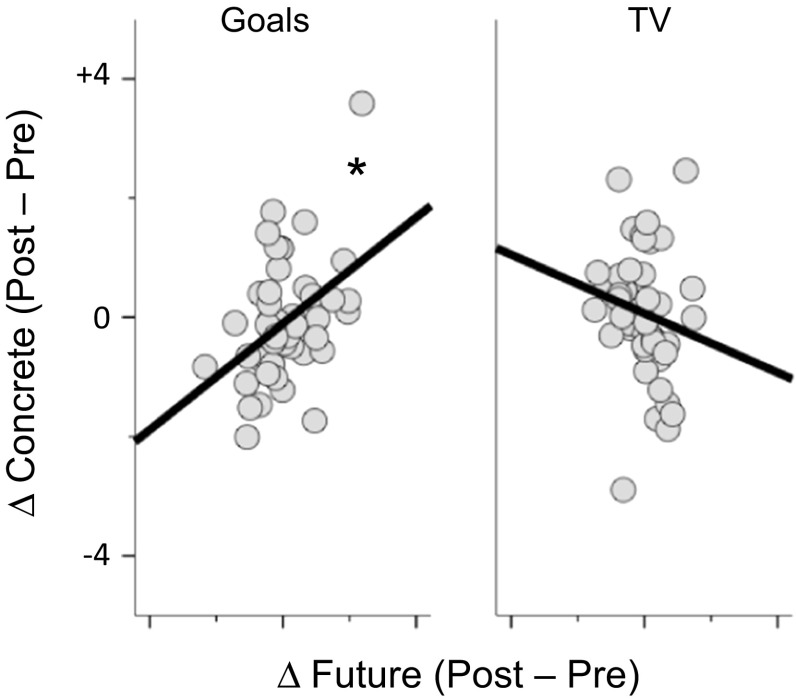
Identification of the relationship between changes in experiential characteristics from baseline and the consolidation of goals. Multivariate analysis of variance (MANOVA) identified that future thinking had increased from baseline if participants produced more concrete goals at the end of the session. The difference scores were calculated by subtracting the baseline measures of experience sampling and goals from *dots* on the *scatter plot* represent individual subjects

To understand the relationship between the changes in the descriptions of the goals and the spontaneous thoughts that occurred during the mind-wandering task, we conducted a multivariate analysis of variance (MANOVA). In this analysis, changes in the six components describing the content and form of thoughts were dependent variables, and the four components describing the change in the descriptions were entered as continuous independent variables. We also included the condition (goal/TV programmes) as a categorical factor. We modelled the main effects of each factor and allowed each component to interact with the experimental condition. This MANOVA indicated a condition by concrete detail interaction [*F* (6, 84) = 2.7, *p* < 0.05] that was due to an association between thoughts about the self and future in the goal condition (*r* = 0.42, *p* < 0.005, 95 % CI lower = .10, upper = .65), but not the TV program condition (*r* = −0.16, *p* > 0.2, 95 % CI lower = −.47, upper = .16). Thus, when an opportunity for spontaneous thought led to an increase in the concreteness of personal goals, this change was related to increases in thoughts regarding the future across this period. Table 4 presents the zero-order correlations between the change in each type of spontaneous thought and each element of the written descriptions, and Fig. 3 illustrates the significant relationship between increasingly concrete descriptions of goals with future thinking. To assess whether this pattern replicated across the samples of participants who did and did not participate in the initial resting-state study, we calculated the correlation between increases in spontaneous future thinking and the changes in concrete goals separately in the behavioural and fMRI samples. This identified a similar pattern in both groups (behaviour only, *r* = 0.38, *p* = 0.06, fMRI, *r* = 0.44, *p* < 0.05).

**Table 4:** Zero-order correlations between the change in self-generated thought and in descriptions of goals and TV programmes

	Future	Past	Task	Images	Detail	Intrusive
*Entire sample*						
C1	0.18	−0.07	−0.15	0.05	0.11	0.08
C2	0.12	−0.01	−0.05	0.04	0.11	−0.04
C3	−0.02	−0.18	0.04	−0.05	0.12	−0.07
C4	−0.04	−0.07	0.10	−0.07	0.24	−0.14
*Goals*						
C1	0.42**	−0.08	−0.07	0.12	−0.04	0.17
C2	0.14	0.00	−0.16	0.07	0.06	0.02
C3	−0.08	−0.19	−0.08	−0.16	0.27*	−0.16
C4	−0.08	−0.11	0.21	−0.07	0.25	−0.11
*TV programmes*						
C1	−0.16	−0.05	−0.23	0.00	0.27	0.00
C2	0.09	−0.02	0.03	0.03	0.15	−0.09
C3	0.13	−0.16	0.15	0.07	−0.11	0.08
C4	0.07	0.01	−0.05	−0.09	0.24	−0.17

C1, concrete; C2, self; C3, negative; C4, past

* Correlation is significant at the 0.05 level (2-tailed)

** Correlation is significant at the 0.01 level (2-tailed)

Finally, we explored whether the pattern linking change in the concreteness of goals was related to low levels of future thought in the baseline session, in the post-description session or both. We conducted two separate linear regressions with the change in concreteness as the dependent variable and the loading on future thinking in sessions 1 and 2 as independent variables. This model was run separately on the participants who participated in the goal and in the TV programme conditions. This revealed a significant model for the goals condition [*F* (2, 48) = 5.02, *p* < .01] but not in the TV programmes condition [*F* (2, 45) = .761, *p* = .47]. In the goal condition, the largest changes in the concreteness were associated with lower levels of future thinking in session 1 and higher levels in session 2 (see Table 5). This pattern indicates that increasingly concrete goals were produced by individuals who produced low levels of future-related thoughts but subsequently increased these reports in the second session.

**Table 5:** Results of linear regressions exploring the relationship between the change in concreteness of descriptions and future thinking in sessions 1 and 2

	*B*	SE	*β*	*t*	*p*
*Goal*					
Constant	−0.32	0.15		−2.14	0.04
Future session 1	−0.60	0.22	−0.42	−2.72	0.01
Future session 2	0.66	0.23	0.44	2.84	0.01
*TV programme*					
Constant	0.19	0.18		1.06	0.30
Future session 1	0.41	0.33	0.28	1.21	0.23
Future session 2	−0.28	0.34	−0.19	−0.81	0.42

### Relation to functional connectivity

**Fig. 4:**
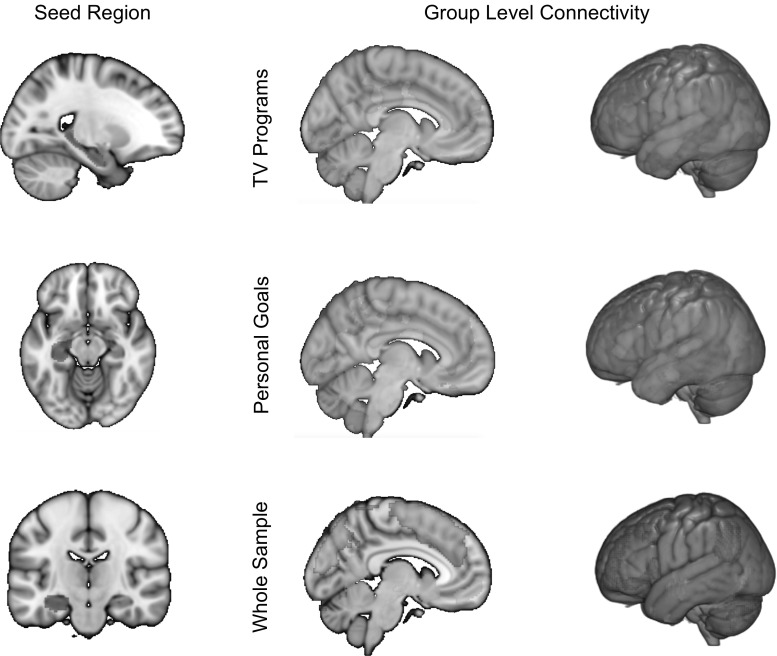
Examination of the relationship between the resting-state connectivity of the hippocampus and its relation to changes in the concreteness of goals. Functional connectivity from the left hippocampus yielded comparable patterns of connectivity across the goals and TV program condition (*upper panel*) indicating no differences in hippocampal connectivity across experimental groups. Analysis of the group-level connectivity patterns indicated coupling between the hippocampus with limbic regions, aspects of medial pre-frontal and cingulate cortex and aspects of lateral temporal–occipital cortex

Having identified that spontaneous thoughts about the future increase when an opportunity to engage in spontaneous thought leads to changes in the concreteness of personal goals, we next assessed whether these changes in concreteness were predicted by individual differences in the pattern of hippocampal functional connectivity assessed during the resting state. We first verified that the overall patterns of hippocampal connectivity were consistent across the two groups. The upper panel of Fig. 4 presents unthresholded connectivity maps for the left hippocampus seed separated into the participants who were subsequently allocated to the goals or the TV programme condition, and it is apparent that there were no gross differences in functional connectivity across the participants assigned to the two conditions. We compared these groups using FMRIB’s Local Analysis of Mixed Effects (FLAME) with cluster-forming threshold of *Z* = 2.3 and corrected for multiple comparisons with an FWE level of *p* < .05. This analysis found no differences in the group maps and revealed clusters of significant functional connectivity within subcortical regions, in medial pre-frontal and posterior cingulate cortex, lateral regions of the temporal lobe and elements of anterior lateral occipital cortex (see Fig. 4, lower panel). The significant clusters are presented in Table 6.

**Table 6:** Spatial cluster identified in the group-level analysis of hippocampal connectivity

Region	Extent	*p*	Centre of gravity		
	Voxels		*X*	*Y*	*Z*
*Positive map*					
Limbic	28,464	0.000	−4	−24	−15
Prefrontal	602	0.041	−1	49	−13
*Negative map*					
Mid-cingulate	23,572	0.000	13	5	33
Cerebellum	2116	0.000	−32	−71	−38
Inferior parietal	1732	0.000	−49	−57	35
Posterior temporal (right)	733	0.017	61	−32	−15

**Fig. 5:**
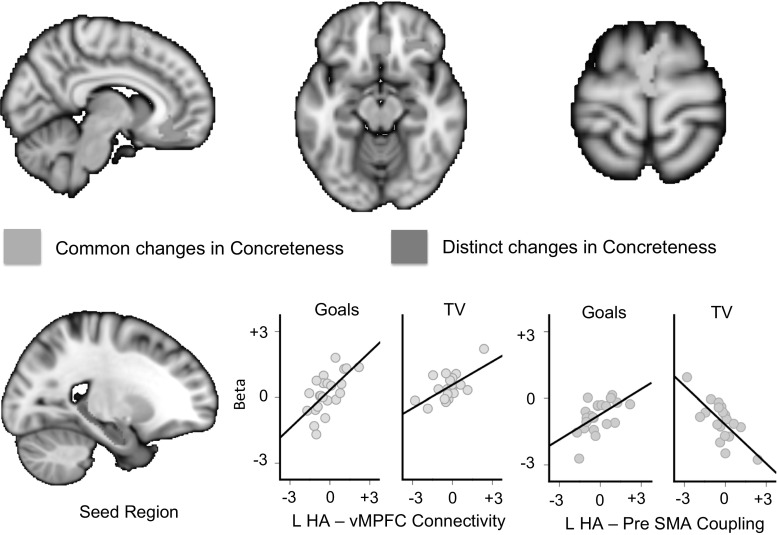
Relation between hippocampal connectivity and changes in concreteness of descriptions of goals versus TV programmes. Functional connectivity between the left hippocampus and the medial pre-frontal cortex was a characteristic of individuals who tended to generally increase the level of concrete details in their descriptions regardless of whether they were goals or TV programmes. We also found that patterns of positive connectivity between the left hippocampus and the pre-SMA were associated with increases in concreteness in the goals condition, whereas it was associated with reductions in concreteness in the TV program condition

Having demonstrated that the experimental groups did not differ in their general patterns of hippocampal connectivity, we conducted a group-level regression examining how these connectivity patterns predicted changes in concreteness that were common to the goals and TV condition. In this model, the independent variable was the components describing the change in concreteness. We used a cluster-forming threshold of *Z* = 2.3, and the subsequent spatial maps were corrected for multiple comparisons with an FWE level of *p* < .05. We first explored individual differences in functional connectivity that varied with increases in the concreteness of descriptions in both conditions (i.e. an association between changes in concreteness and functional connectivity that was common to both the goals and the TV programme conditions). This identified a cluster in the ventromedial pre-frontal cortex, centred on MNI coordinates: 8, 35, −14, which was 5880 mm^3^ in volume (see Fig. 5, upper panel). Next, we examined individual differences in connectivity that were associated with greater changes in concreteness for descriptions of goals rather than TV programmes (i.e. specific patterns of connectivity that were greater for participants who increased the concreteness in their descriptions of goals more than for TV programmes). This contrast identified a single cluster in the dorsal medial pre-frontal cortex extending into the pre-SMA: this was centred on MNI coordinates 5, 3, 64, with an approximate volume of 4880 mm^3^ (see Fig. 5, upper panel).^2^

To facilitate the interpretation of these patterns of connectivity, we extracted the *β* values from both spatial clusters for each participant and plotted them against their change in concreteness, separately for each condition. It can be seen in Fig. 5 that while increases in concreteness in both conditions were associated with greater coupling between the hippocampus and the cluster in the ventromedial pre-frontal cortex, the coupling with the pre-SMA cluster was associated with more concrete descriptions in the goal condition (*r* = .60, *p* < .001 95 % CI lower = .315, upper = .799] but less concrete descriptions of television programs (*r* = −.68, *p* < .001 95 % CI lower = −.88, upper = −.22). Given the relatively small sample size, we performed a spilt-half reliability check in both cases this revealed acceptable levels of consistency (Spearman–Brown: goals = .75, TV programs = .80). Thus, our data show that coupling between the hippocampus and the ventromedial pre-frontal cortex is predictive of increased concreteness for both descriptions of goals and TV programmes, while an extensive cluster in the dorsomedial frontal cortex was uniquely associated with the tendency to increase the concreteness of personal goals.

Finally, to better understand the psychological significance of this pattern of functional coupling, we conducted a post hoc analysis targeted at determining whether the pattern of functional coupling was related to less concrete descriptions of goals in the initial session, or higher levels in the second session. We conducted a multiple regression in which the dependent variable was the hippocampal–pre-SMA coupling and the independent variables were the raw ratings of abstract and concreteness in the descriptions gained in the first and second sessions. We conducted these separately for the goal and TV programs conditions. In both cases, this produced a model which was significant [goals, *F* (4, 22) = 4.41, *p* < .01; TV program, *F* (4, 18) = 5.2, *p* < .01, see Table 7]. In the case of the description of goals, we found a reduction in connectivity between the hippocampus and the pre-SMA associated with more abstract goals at in session 1 and a reduction in abstraction in session 2. In the TV programme condition, we observed that the reverse connectivity between the hippocampus and the pre-SMA was associated with less abstraction in session 1 and more abstraction in session 2.

**Table 7:** Relation between hippocampal–pre-SMA coupling and ratings of descriptions as concrete or abstract in each session

	*B*	SE	*β*	*t*	*p*
*Goal*					
Constant	−3.67	2.43		−1.51	0.15
Abstract session 1	1.66	0.52	1.90	3.21	0.01
Concrete session 1	0.78	0.50	0.82	1.58	0.13
Abstract session 2	−1.16	0.46	−1.20	−2.55	0.02
Concrete session 2	−0.33	0.44	−0.30	−0.75	0.46
*TV programmes*					
Constant	−3.46	2.32		−1.49	0.16
Abstract session 1	−0.91	0.39	−0.65	−2.32	0.04
Concrete session 1	0.31	0.49	0.19	0.63	0.54
Abstract session 2	1.44	0.46	0.82	3.12	0.01
Concrete session 2	−0.10	0.47	−0.06	−0.21	0.84

## Discussion

Our experiment sets out to determine the experiential and neural correlates of the process through which we spontaneously refine the steps to achieve personal goals. We found a reliable pattern of correlation, indicating that participants who developed more concrete goals had engaged in greater spontaneous future thinking between describing their goals for the first time and re-describing them. That is future thinking did not increase as a consequence of writing about goals, it only increased for those participants who would subsequently produce more concrete descriptions of goals. Importantly, increases in concreteness were not associated with changes in other aspects of spontaneous thought, indicating that thoughts about the future were the precedent of more specific written descriptions of goals. Prior work has shown that specific plans lead to more effective goal achievement (Gollwitzer and Sheeran 2006) and that more concrete goals have a protective value with respect to certain features of affective dysfunction (Watkins 2008). In this context, our results are important because they illustrate one way in which the self-generated thoughts in the mind-wandering state can have adaptive qualities—allowing our plans for the future to become better specified (for further discussion of the costs and benefits of mind-wandering, see Mooneyham and Schooler 2013). The results, therefore, provide a mechanism that can explain why this style of future thinking can lead to improvements in mood (Ruby et al. 2013) and reductions in the physiological responses to stressors (Engert et al. 2014).

Our resting-state functional MRI findings highlight that our capacity to develop more concrete descriptions of both goals and aspects of our knowledge (of TV programmes) is supported by brain networks centred on the hippocampus. We observed stronger coupling between the hippocampus and the ventromedial pre-frontal cortex in individuals who produced more concrete details in their descriptions of either TV programmes or goals. This cluster is within the connectivity map of the hippocampus itself, suggesting that together these two regions form a large-scale network that is generally important in embellishing information in memory. Our data suggest that communication between these regions reflects a general process through which explicit descriptions are made concrete, a conclusion that is consistent with recent evidence implicating the hippocampus and the ventromedial pre-frontal cortex in contributing conceptual, or schematic, knowledge to episodic memories (Kumaran et al. 2009; Bonnici et al. 2012; Zeithamova et al. 2012; van Kesteren et al. 2013).

In contrast, greater coupling between the hippocampus and more dorsal medial frontal regions, including the pre-SMA, was a specific predictor of the generation of more concrete goals. This may represent a mechanism that allows an individual to mentally simulate possible paths to goal achievement. The pre-SMA has a known role in planning actions (Kennerley et al. 2004), and a rostral region adjacent to our cluster is important in the process through which participants choose actions which are minimally constrained by the environment (Nachev et al. 2005). Importantly, several recent studies have extended the role of the pre-SMA beyond motor planning by implicating it, as well as the hippocampus, in the generation of detailed personal goals (Spreng et al. 2015) as well as in the ordering of future events (D’Argembeau et al. 2015). Our data build on these observations by illustrating the role that future thought during the mind-wandering state plays in this process. We speculate that connectivity between the hippocampus and the pre-SMA may be important in devising more concrete descriptions of personal goals because together they would support a cyclical process in which goal states are evaluated, refined and encoded for subsequent evaluation. It is possible that the integrity of this cycle could depend on the role of the hippocampus in *pattern separation* since the iterative process by which imagined goals become more concrete requires that more refined goal steps are not confused with the less concrete goals upon which they are based. An alternative possibility is that the connectivity between the hippocampus and the pre-SMA reflects a state of uncertainty that is associated with reduced future thinking during session 1, as well as personal goals that are only weakly specified. These two views could be easily dissociated by a study that measures neural functioning online during the period when personal goals are consolidated. We recommend that future investigations measure neural processes online during the period when personal goals become refined to identify the specific mechanism through which the hippocampus contributes to the consolidation of goals.

There are a number of important limitations associated with the current study. First, we did not record BOLD activity in the period following the initial descriptions of goals, and although we expect coupling between the hippocampus, the vMPFC and the pre-SMA to be important during this period, the specific pattern of activity that occurs in the period when goals become more concrete remains an open question. Second, although our findings show a robust link between future thinking and more concrete personal goals, the smaller sample in the brain-imaging component of this investigation means that these data should be treated with caution. In particular, although the stronger coupling with the vMPFC was true of the entire sample, the specific coupling between the hippocampus and the pre-SMA in the smaller goals condition means that this result has greater chance of being a type II error. We recommend replication of this effect with a larger sample. Third, although our study shows that descriptions of goals changed in tandem with the extent of future-related thinking, it is possible that other aspects of memory would change in a similar manner. Future work should consider whether future thinking, as well as other types of self-generated thought, acts to concretize other aspects of experience (such as past achievements). Fourth, our control condition (TV programmes) varies from the personal goals on many variables. Thus, although our data implicate future thinking, rather than other aspects of experience during the mind-wandering state, in the process of goal consolidation, it is unclear which aspect of goal representation this phenomenon reflects (e.g. elements of the future or the prioritisation of aspects of task processing). Future work could compare the role of future thinking in the consolidation of goals with aspects of the future that have a less direct relationship to action. Finally, some of the reliability for the coding scores, in particular the concrete dimension, was quite low. Future studies should therefore explore ways of coding goal transcripts which are more reliable.

Overall, our study demonstrates that the tendency to consider the future during mind-wandering is associated with the development of personal goals, making them more concrete even in the absence of the chance to act. The strength of this process was related to individual differences in the functional connectivity of the hippocampus with the pre-SMA at rest, providing insights into the neural architecture that might support the transformation of abstract goals into more concrete plans using imagination. Since prospective thought is a common form of thinking during the mind-wandering state, these results highlight the important role that future thought plays in our lives by helping us make decisions about what to do and how to do it.

## Footnotes

1 In this decomposition, the weighting for C1 was negatively weighted on concreteness and positively related to abstractness. For simplicity of explanation, we present the reversed factor loading for this component in all subsequent analysis in which a positive score indicates that written descriptions became more concrete.

2 Using the right hippocampus as a seed region, we found a similar pattern of increased connectivity with the medial prefrontal cortex associated with greater concreteness in descriptions of either TV programmes or goals. This cluster was centred on MNI coordinates 6, 35 −4 and was approximately 4800 mm^3.^
